# Point of care ultrasound assessment of the optic nerve sheath diameter in critically ill children

**DOI:** 10.1186/s12887-025-05798-z

**Published:** 2025-06-27

**Authors:** Ahmed A. EL-Nawawy, Ahmed A. El Beheiry, Aya M. Abdelaziz, Hadir M. Hassouna

**Affiliations:** 1 Department of Pediatrics, Faculty of Medicine, Alexandria University, Alexandria, Egypt; 2 Department of Radiolody, Faculty of Medicine, Alexandria University, Alexandria , Egypt

**Keywords:** Critically ill children, Intracranial pressure, Intensive care unit, Non-invasive monitoring, Optic nerve sheath diameter, Point-of- care ultrasound

## Abstract

**Background:**

Raised intracranial pressure (ICP) is a typical neurological problem in critically ill children, which is associated with poor clinical outcomes or even death. The purpose of the study was to evaluate the efficacy of optic nerve sheath diameter (ONSD) as a point-of-care testing in the pediatric intensive care units for early diagnosis of raised intracranial pressure and as a follow up tool for treatment response.

**Method:**

A prospective observational study was conducted in the pediatric intensive care unit of a tertiary care children's hospital. Consecutive children aged one month to twelve years with Glasgow Coma Scale less than or equal to 8 were included. Brain CT was performed just before or within few hours of admission to identify raised ICP. Two trained examiners in the same work place, who were blinded to the clinical details of the patients, performed the ONSD sonography concurrently. The ONSD was measured in the left and right eyes on admission, after 1 h, after 12 h, and after 24 h.

**Results:**

Forty-two patients were categorized into 29 children with raised ICP and 13 children with non-raised ICP. The ONSD was significantly higher in the raised ICP group at all times in both eyes. It showed a significant decrease over time in both groups. A cutoff value of ≥ 4.3 mm was found to be an acceptable discriminator of ICP with Area Under the ROC curve (AUC) = 0.788 (95% CI 0.740- 0.830) (*p* < 0.0001), sensitivity of 59.91% (95% CI: 53.30–66.27) and specificity of 83.65% (95% CI: 75.12–90.18).

**Conclusions:**

In critically ill children with non-traumatic causes of raised ICP, point of care ultrasound of ONSD shows a good diagnostic test accuracy for early diagnosis of raised ICP as well as follow up tool of treatment response if used serially. In addition, it is very good at correctly identifying individuals who do not have raised ICP to avoid unnecessary interventions.

## Background

Raised intracranial pressure (ICP) is a typical neurological problem in seriously ill children, which is associated with poor clinical outcomes or even death [1, 2]. Finding the underlying reason and taking appropriate action to lower ICP are the primary goals for patients who present with elevated ICP. In cases where determining the underlying problem may require some time, it is crucial to lower ICP promptly [3]. Although invasive ICP monitoring is still the gold standard for measuring ICP, it is not regularly carried out due to the lack of available neurosurgeons and contraindications including thrombocythemia or coagulopathy. Furthermore, problems like bleeding and bacterial colonization may make invasive ICP monitoring less feasible [4, 5]. In spite of being non-invasive, both CT and MRI need transportation across multiple institutions, which may be challenging, particularly if the child is on mechanical ventilation [2, 6]. Consequently, a non-invasive, reproducible, and simple bedside technique for the evaluation of raised ICP is crucially needed. Recently, ocular ultrasonography through measuring optic nerve sheath diameter (ONSD) has shown promise in identifying raised ICP even prior to the development of papilledema, which takes several hours to show up [7, 8]. The optic nerve sheath is derived from the three layers of the meninges, and the pressure change in the intra-orbital subarachnoid space is the same as that of the intracranial subarachnoid space. When ICP increases, the optic nerve sheath expands and thickens [8, 9]. The ONSD in adults has been extensively studied and certain cutoff points have been established. However, the pediatric population has not been sufficiently studied especially in non-traumatic non-surgical children [10]. For that reason, the current study was set out to evaluate the efficacy of measuring ONSD as a point-of-care testing for diagnosis of elevated ICP as well as monitoring of treatment response in critically ill children particularly in settings where invasive monitoring is not feasible or available.

## Methods

A prospective observational study was conducted in the Pediatric Intensive Care Unit of a tertiary care teaching hospital from the 1 st of November 2022 to the 31 st of August 2023. Consecutive children aged one month to twelve years with Glasgow Coma Scale less than or equal to 8 were included. Children whose brain computed tomography (CT) could not be performed due to their medical condition were excluded. We excluded children having any contraindication for optic nerve sonography as in possible global rupture or retrobulbar bleeding, open ocular trauma, or periorbital wounds. Patients deceased within 24 h of admission were not included. Patients with ≥ 0.3 mm inter-observer variability measurements were also excluded.

Brain CT was performed using a Simens® AS 16 Slice CT Scanner (Simens®, Saint Paul, MN, USA) just before or within few hours of admission to PICU. CT images were reviewed by the consultant radiologist who was blinded to both clinical and ONSD measures. Raised ICP was identified by presence of effacement of the ventricles, basal cisterns and other CSF spaces, brain herniation, midline shift and loss of grey-white matter differentiation. Accordingly, patients were categorized into raised ICP group and non-raised ICP group.

### ONSD measurement

Two trainedexaminers in the same work place, who were blinded to the clinical details of the patients, performed the ONSD sonography concurrently. Each examiner independently measured each optic nerve in real time and the measurements were confirmed by the master trainer off-line. The final value was the average for both values. Interobserver variability was reported and ≥ 0.3 mm inter-observer variability measurements were excluded. The measurement of ONSD was conducted using a SonoSite™ M-Turbo point-of-care ultrasound machine (SonoSite® Inc., Bothell, WA, USA) using a high-resolution L25 linear array transducer with a frequency of 7.5 MHz. The L-shaped probe had a small footprint providing better sonographic contact with the eye. An ocular preset was used with low mechanical index values (MI < 0.23), a limit of thermal index (TI) ≤ 1, and an intensity threshold of ≤ 50 mW/cm2 to achieve maximum patient safety following the “As Low As Reasonably Achievable (ALARA) principle” without sacrificing the examination's diagnostic efficacy [11]. ONSD was measured on admission, after one, 12, and 24 h of admission.

**Fig. 1:**
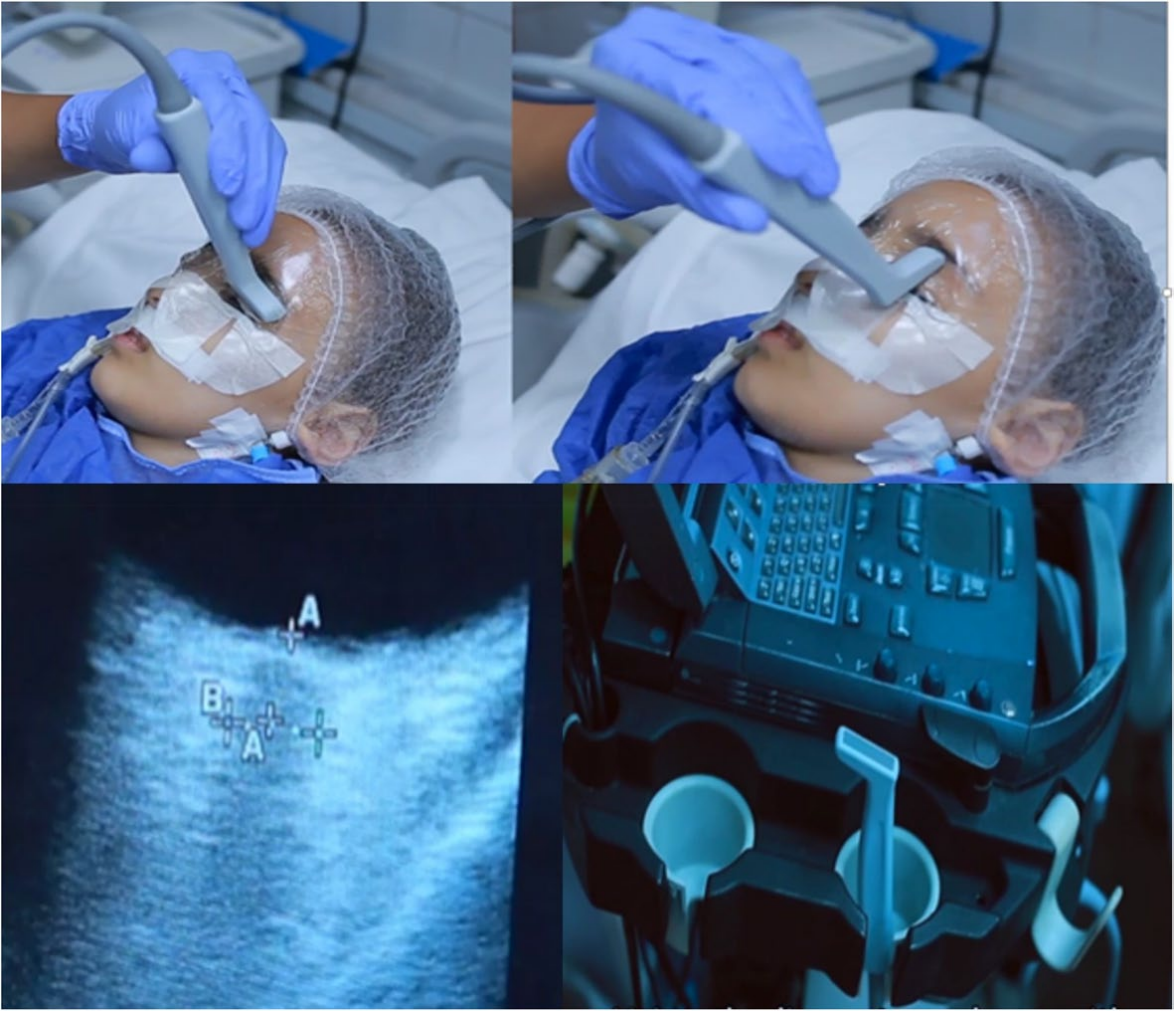
Study technique, the device used and the image obtained. (These images were obtained after consent from the patient’s parents for publication)

The patients were placed supine, with a centralized head, and the upper body was elevated at a 30° angle. A small coating of gel was applied to both eyelids of closed eyes covered with an adhesive transparent strip. Care was taken to avoid air entrapment between the film and external eyelids, as this would degrade the image quality. Using B mode, the linear transducer was placed on the eyelid and gently pressed on the eyeball. By moving the probe over the closed upper lid, two measurements were taken for each optic nerve in the vertical and transverse planes. A cross-sectional image of the globe and the retrobulbar region's components was obtained. The globe of the eye is the prominent feature in the anterior orbit with its anechoic vitreous compartment and echogenic optic disc at its posterior wall [7, 12, 13]. The ultrasonic gain and output intensity were modified to achieve the maximum contrast between the hypoechoic optic nerve complex and the echogenic retrobulbar fat after the beam was focused on the post-bulbar region. The latter was seen as a homogeneous, well-defined low-reflection zone that extended posteriorly from the bulb's base [7]. The optic nerve sheath appeared as a bilateral, lateral, narrow hypoechoic line that went parallel to the nerve [6, 7, 13]. In order to demonstrate the optic nerve in the axial plane as required by the ideal method, patients who had not fixed on a primary gaze needed to have their angulation slightly adjusted then cursors were placed on the optic nerve complex's inner outlines, 3 mm posterior to the optic disc (the location where the sheath is thinnest and most readily expands in response to a rise in intracranial pressure) [6, 7]. The horizontal distance between the two cursors, which was perpendicular to the scanning vertical axis, was used to determine the ONSD [6, 7, 13] (Fig. 1).

### Fundus examination

As there is a strong association between increased ICP and presence of papilledema, a funduscopic examination was done for those patients to detect presence of papilledema or any retinal changes on admission, after one, 12 and 24 h from admission. A training session on the ophthalmoscope was carried out in the PICU under the direction of a senior ophthalmologist over a period of one month. The examinations were done using a Keeler® Jazz direct ophthalmoscope (Keeler®, Phoenixville Pike Pennsylvania, USA).

### Quality assessment

Quality assessments were evaluated by using the Quality Assessment of Diagnostic Accuracy Studies (QUADAS-2) tool and the Risk of Bias table in Review Manager 5.3® software. It was performed by two eminent pediatric professors [14].

### Statistical methods

The statistical analysis was performed by an expert statistician. Data were collected and analyzed using the Statistical Package for Social Science (SPSS) program (version 25) [15]. Kolmogorov–Smirnov test of normality of the distribution of the variables revealed significance, so the non-parametric statistics was adopted [16]. Comparisons were carried out between two studied independent abnormally distributed subgroups using Mann–Whitney U test. Repeated measures analysis was carried out using Friedman test. Post-hoc pair-wise comparisons, when Friedman test was significant, were carried out using Dunn- Sidak test for multiple comparisons. Significance levels (p-values) have been adjusted by the Bonferroni correction for multiple tests. Z-test for comparison of two independent proportions was used. Non-parametric Kendall's tau correlation (τ) was used. Area under the ROC (AUC) was carried using MedCalc Software version 14. Youden index was used to determine the best cut-off value. Nomogram for calculations of properties of distributions was done using online application [17]. The sample size was calculated based on a previous study aimed to provide clinical strategies that will enhance clinicians'assessment of bilateral ONSD "Mehrpour M, Oliaee Torshizi F, Esmaeeli S, Taghipour S, Abdollahi S. Optic nerve sonography in the diagnostic evaluation of pseudopapilledema and raised intracranial pressure: a cross-sectional study. Neurol Res Int. 2015;2015:146,059." They found a close correlation between ONSD on ocular ultrasound and raised ICP. Based on their results, a total sample size of 38 patients was enough required sample to conduct the diagnostic test accuracy study with a minimum sample size of 19 patients for evidence of increased ICP, using a significance level of 95% (α = 0.05) to provide a study power of 80%. The sample size was computed using Medcalc statistical software (version 14.8.1.)

## Results

**Fig. 2:**
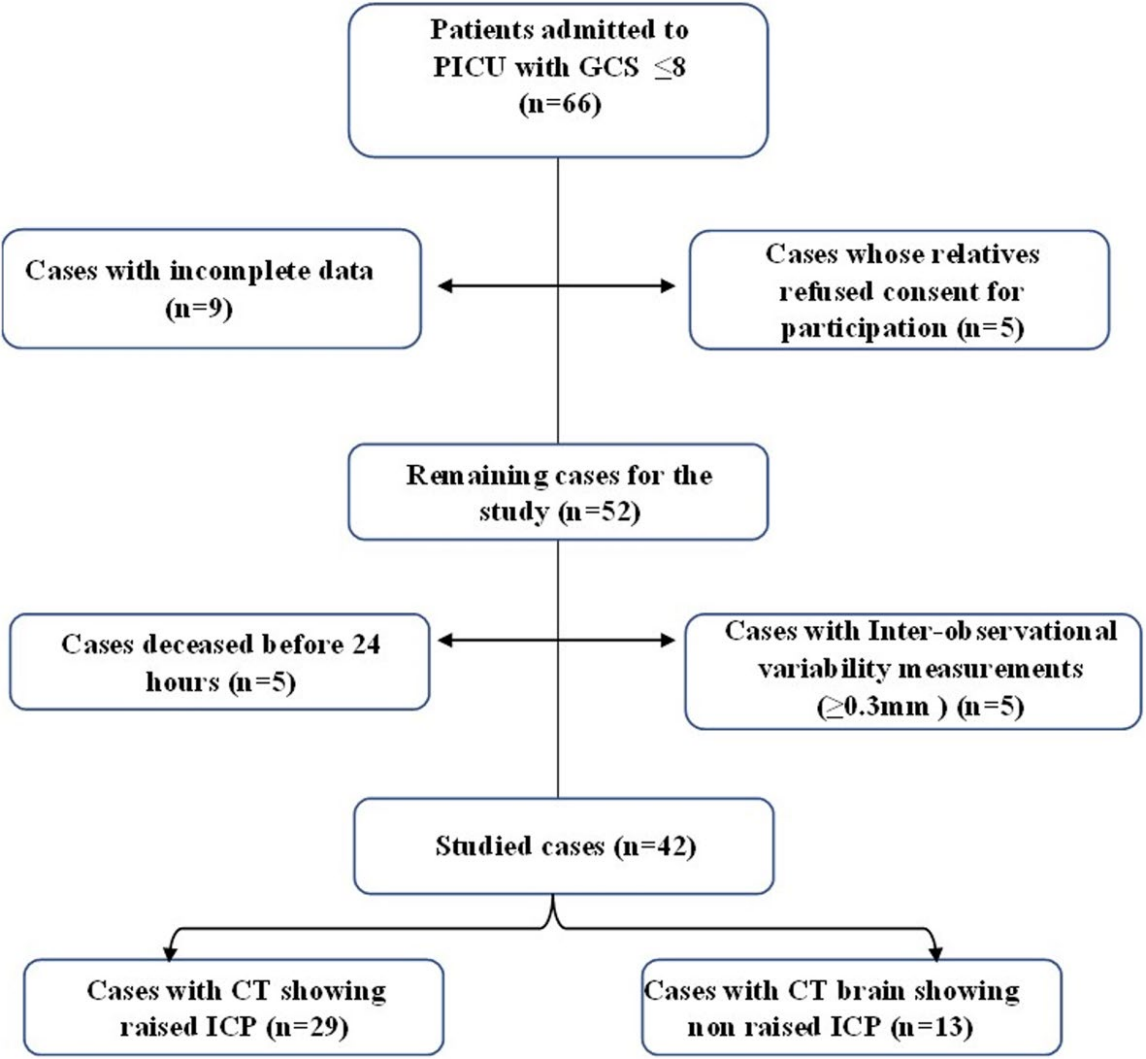
Flow diagram of the study

Sixty-six patients having Glasgow Coma Scale less than or equal to 8 were admitted to PICU during the study period. Twenty-four patients were excluded per the exclusion criteria. Forty-two patients were included and categorized into 2 groups according to the CT brain findings as shown in the study flow diagram (Fig. 2).

Demographic data, baseline characteristics, and mortality of both groups are presented in Table 1. There was a significantly higher partial pressure of carbon dioxide (PaCO2) in the raised ICP group compared with the non-raised ICP group (*p* = 0.0467). While elevated PaCO₂ can influence ICP, in this case, the increased ICP is more likely due to the primary disease process, given that PaCO₂ is typically well-controlled in mechanically ventilated patients. The raised ICP group had significantly lower heart rate and significantly higher mean arterial blood pressure compared with the non-raised ICP group among most age groups explained by the Cushing reflex which is the physiological response of the nervous system to acute increase intracranial pressure. There was no statistical difference among the 2 groups as regard the fate.

**Table 1:** Demographic and baseline data

	Raised intracranial pressure group (*n* = 29)	Non-raised intracranial pressure group (*n* = 13)	*P* value
Age (years)^a^	1.58(0.5–4)	3(0.5 ± 5)	MW *p* = 0.406
Sex (*n*%)			X^2^ *p* = 0.335
Male	18(62.07%)	6(46.15%)	
Female	11(37.93%)	7(37.93%)	
PELOD-2^a^	4(1–6)	2(1–4)	MW *p* = 0.136
PIM-3^a^	6 (2.3–16)	9(2.1–13.2)	MW *p* = 0.957
Oxygen Saturation (%)|^a^	99(98–100)	100(99–100)	MW *p* = 0.373
PaCO₂ (mmHg)^a^	51(48–63)	35(31–48)	MW *p* = 0.0467*
Heart Rate (beats/min)^a^	103(95–114)	118 (103–134)	MW *p* = 0.044*
Mean arterial blood pressure (mmHg)^a^	72(77–79)	60(66–77)	MW *p* = 0.025*
Temperature (◦C)^a^	37.3(37–39)	37.2(37–38)	MW *p* = 0.891
Respiratory Rate (breaths/min)^a^	29(20–37)	24(25–29)	MW *p* = 0.149
Mortality (*n*%)	10(34.48%)	7(53.85%)	X^2^ *p* = 0.237

*PELOD-2* Paediatric Logistic Organ Dysfunction 2 Score, *PIM-3* Paediatric Index of Mortality 3, *MW p* *p*-value of Mann Whitney test, *X*^*2*^*p p*-value of Pearson Chi-square test

^a^median (IQR)

*Statistically significant

This study included 29 children with raised intracranial pressure, 12/29 (41.37%) of the children had CNS infections, 8/29 (27.68%) had status epilepticus, 1/29 (3.44%) had aplastic anemia, 6/29 (20.68%) had septic shock and 2/29 (6.89%) had other diagnoses as an inborn error of metabolism or hypernatremic dehydration. In addition, this study included 13 children with non-raised intracranial pressure, 3/13 (23.07%) of the children had CNS infections, 2/13 (15.48%) had status epilepticus, 8/13 (61.53%) had septic shock.

CT was performed for all patients at a mean time of 1.35 h before admission and 2.21 h after admission. Signs of increased intracranial pressure in the raised ICP group were midline shift in 10/29 (34.48%), effacement of ventricles and sulci in 13/29(44.83%), effacement of basal cisterns in 3/29(10.34%), brain herniation in 2/29 (6.89%) and 4/29 (13.79%) had hydrocephalus.

**Fig. 3:**
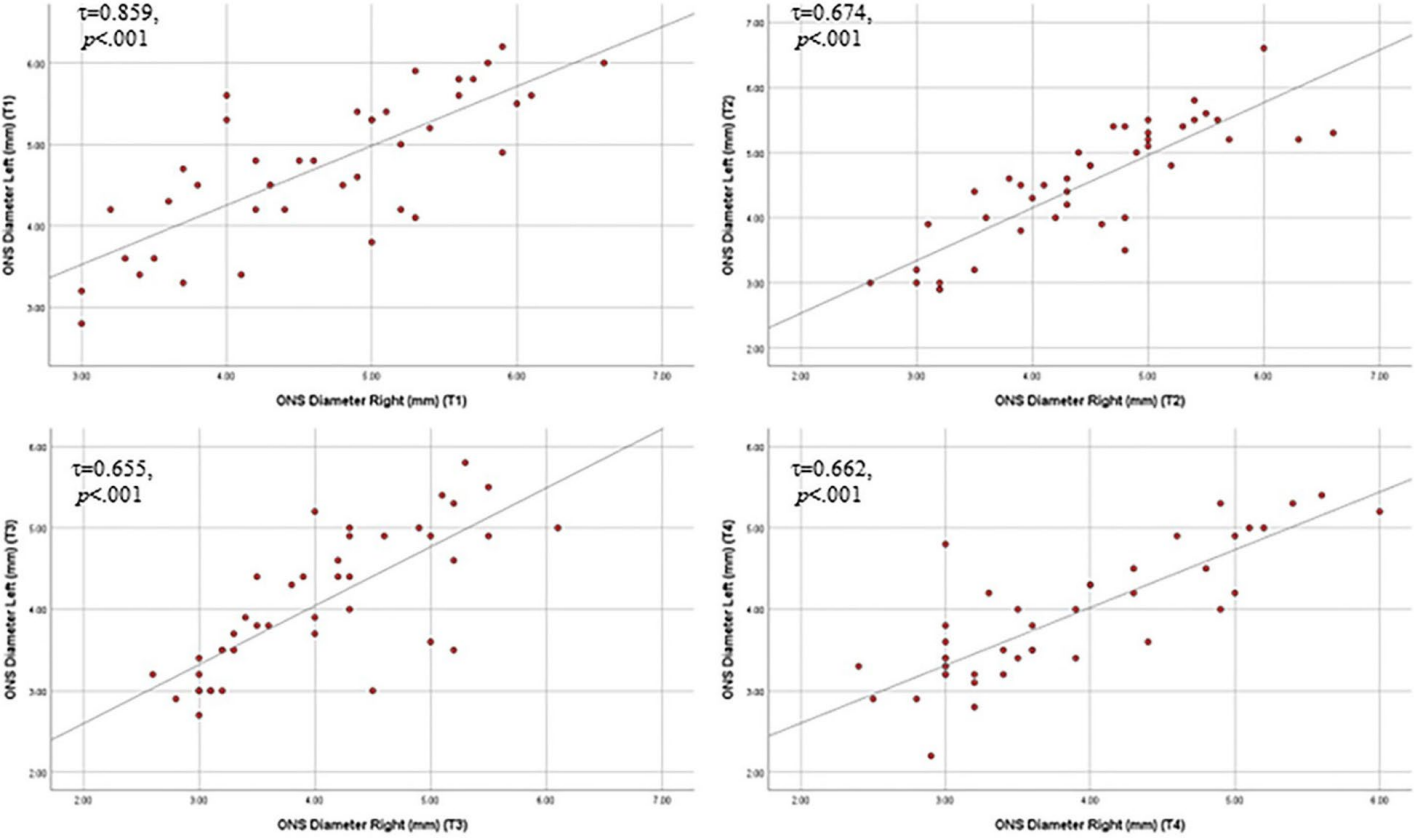
Binocular correlation of ONSD at different times. (T1: on admission, T2: after 1 h, T3: after 12 h, T4: after 24 h of admission)

For both raised and non-raised ICP groups, the ONSD was measured in the left and right eyes on admission (T1), after 1 h (T2), after 12 h (T3), and after 24 h (T4). (Table 2) Binocular correlation of ONSD measurements revealed high positive correlation at T1 (τ = 0.859, *p* < 0.001) and moderate positive correlation at T2, T3 and T4(τ = 0.674, τ = 0.655, τ = 0.66; *p* < 0.001; respectively) (Fig. 3) Both groups showed significant decrease in ONSD over time (*p* = 0.001 for both eyes in both groups).

**Table 2:** ONSD measurements in raised and non-raised groups

	**Left Optic Nerve Sheath Diameter (mm)**			**Right optic nerve sheath diameter(mm)**		
	Raised intracranial pressure group (*n* = 29)	Non- raised intracranial pressure group (*n* = 13)	*P* value	Raised intracranial pressure group (*n* = 29)	Non- raised intracranial pressure group (*n* = 13)	*P* value
T1 (On admission)	5.2 (4.5–5.6)	4.3 (3.6–4.5)	MW *p* = 0.004*	5.2(4.5–5.6)	3.8(3.4–4.3)	MW *p* = < 0.001*
T2 (After 1 h)	5 (4–5.4)	4.2(3–4.6)	MW *p* = 0.005*	4.8(4.3–5.4)	3.8(3.2–4.3)	MW *p* = 0.002*
T3 (After 12 h)	4.4(3–5)	3.4(3–3.8)	MW *p* = 0.003*	4.3(3.5–5.1)	3.1(3–3.6)	MW *p* = < 0.001*
T4 (After 24 h)	4.2 (3.5–4.9)	3.2 (3.2–3.5)	MW *p* = 0.001*	4(3.2–4.9)	3 (3–4)	MW *p* = 0.003*
*p* value	F*p* = < 0.001*	F*p* = < 0.001*		F*p* = < 0.001*	F*p* = < 0.001*	

*T1* on admission, *T2* 1 h after admission, *T3* 12 h after admission, *T4* 24 after admission, data presented in median(IQR), *MW p* p-value of Mann Whitney test, *Fp* *p*-value of Friedman test,

*Statistically significant (*p* ≤ 0.05)

Then, the patients of the raised ICP group were regrouped according to the state of their anterior fontanels (AF) into opened and closed AF subgroups. Comparison between left and right ONSD in opened versus closed AF subgroups at different times is shown in Table 3. In the raised ICP group, fundoscopic examination of both eyes revealed papilledema in 5/29(17.24%) at all times.

**Table 3:** ONSD measurements in opened and closed anterior fontanels subgroups among raised ICP group

	**Left Optic Nerve Sheath Diameter (mm)**			**Right optic nerve sheath diameter(mm)**		
	Opened AF group (*n* = 14)	Closed AF group (*n* = 15)	*P* value	Opened AF group (*n* = 14)	Closed AF group (*n* = 15)	*P* value
T1 (On admission)	4.85 (4.6–5.4)	5.6 (4.2–5.9)	MW *p* = 0.016*	5.05(4.5–5.6)	5.3(4.4–5.8)	MW *p* = 0.0415*
T2 (After 1 h)	4.7(4–5.2)	5.4(4–5.5)	MW *p* = 0.047*	4.5(4.1–5.3)	5(4.7–5.4)	MW *p* = 0.036*
T3 (After 12 h)	4.2(3.5–4.4)	4.9(3.7–5.8)	MW *p* = 0.069	4.3(3.5–4.9)	4.6(3.5–5)	MW *p* = 0.457
T4 (After 24 h)	4 (3.6–4.2)	4.8 (3.4 −5)	MW *p* = 0.155	3.9(3–4.4)	4.6(3.2–5)	MW *p* = 0.255
*p* value	*Fp* = < 0.001*	F*p* = < 0.001*		F*p* = < 0.001*	F*p* = < 0.001*	

*T1* on admission, *T2* 1 h after admission, *T3* 12 h after admission, *T4* 24 after admission, data presented in median (IQR), *AF* anterior fontanel, *MW p* *p*-value of Mann Whitney test, *Fp* *p*-value of Friedman test

*Statistically significant (*p* ≤ 0.05)

**Fig. 4:**
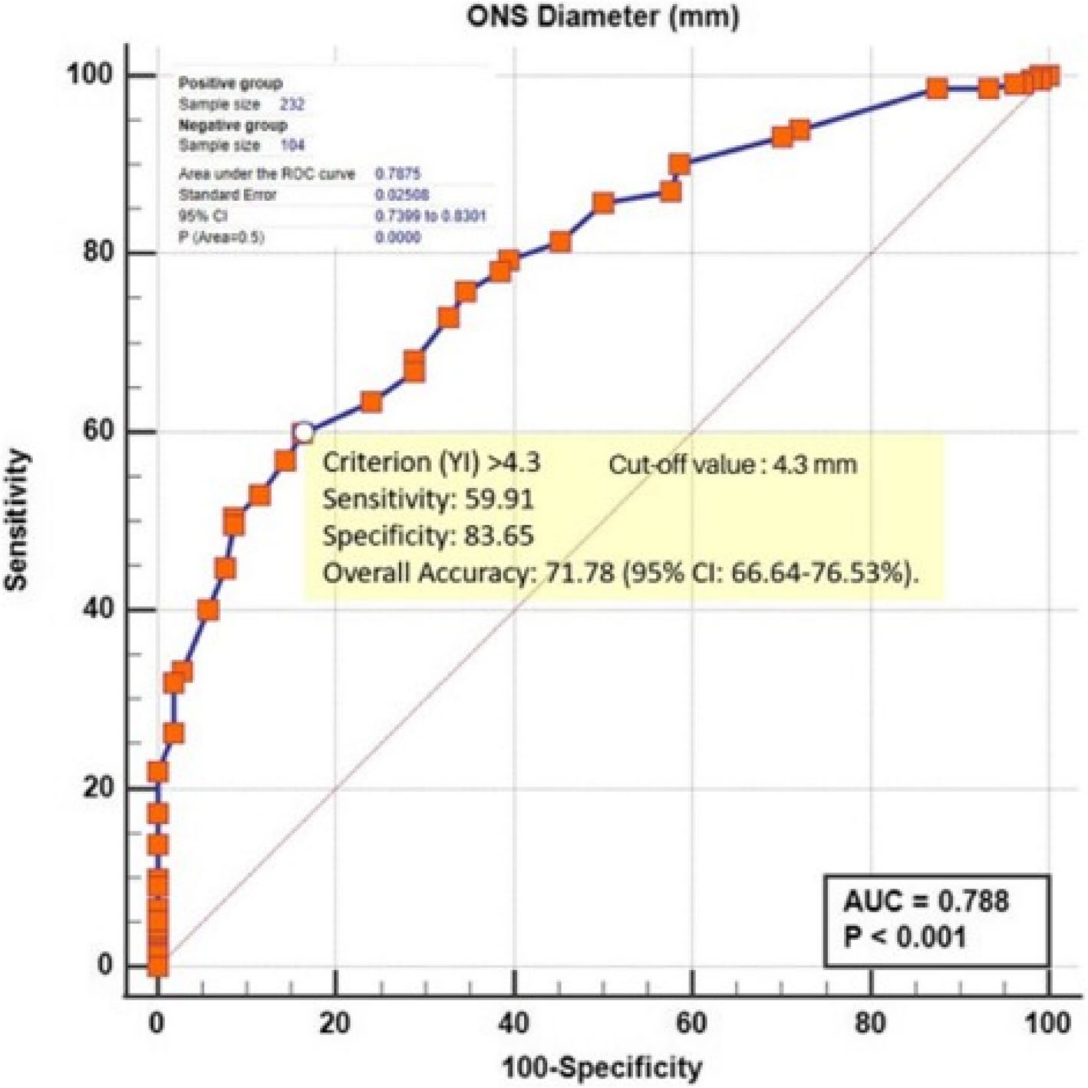
Area under the ROC curve of Optic Nerve Sheath Diameter by Ultrasound as a discriminator for raised intracranial pressure confirmed by Computed Tomography

The ONSD was found to be an acceptable significant discriminator of ICP with Area Under the ROC curve (AUC) = 0.788 (95% CI 0.740- 0.830) (Z = 11.462, *p* < 0.0001). Using Youden index, the diagnostic criterion level is > 4.3 mm with a sensitivity of 59.91% (95% CI: 53.30–66.27), specificity of 83.65% (95% CI: 75.12–90.18), Positive Likelihood Ratio (PLR) of 3.67(95%CI: 2.34–5.73), Negative Likelihood Ratio (NLR) of 0.48(95%CI: 0.4–0.57), Positive Predictive Value (PPV) of 78.57% (95% CI: 70.09–85.15%) and Negative Predictive Value (NPV) of 67.60% (95% CI: 63.57–71.39%, and overall accuracy of 71.78 (95% CI: 66.64–76.53%) (Fig. 4).

Using ONSD of ≥ 4.3 mm as a discriminator of raised ICP revealed 74.13% true positive cases and 65.38% true negative cases. By using the Bayes nomogram, when the ONSD was more than the cut-off value (4.3 mm), the probability of high ICP was increased from 69 to 83%. When the ONSD is less than 4.3 mm, the probability of high ICP was decreased from 69%to 47%.

## Discussion

This study is one of the few studies conducted on children, which focused on the noninvasive monitoring of ICP using point of care sonography of ONSD.

In the current study mortality rate among the rasised ICP group was 34.48%. According to Hou et al. the average mortality rate for patients with elevated ICP was 29.2% [18]. In conclusion, raised ICP may be one of the contributors of poor prognosis and mortality among patients with coma admitted to PICUs.

Invasive ICP monitoring is the gold standard tool for detecting raised ICP. However, it is not feasible due to its invasiveness, high cost, and potential for serious complications such as bleeding or bacterial infection. While most physicians have the opportunity to perform cranial CT in an emergency practice setting, cranial CT consumes time and involves transportation away from the emergency observational room and resuscitation facilities. The current study confirmed the good correlation between ONSD and initial CT scan as regard raised ICP. Jenjitranant et al. conducted a study that verified the association between ONSD and primary CT warning signs of elevated ICP [19]. Legrand et al. stated that except for hydrocephalus and midline shift, which took longer to manifest, all primary CT findings of elevated ICP were linked with larger ONSD [20]. Malayeri et al. as well as Kerscher et al. revealed that children with persistent signs of elevated ICP on CT scans had higher ONSDs [21, 22].

Although ONSD has been well examined in adults and distinct cut-off points have been established, further research in the pediatric population is still required [11]. In the current study, by evaluating ONSD in both eyes of 29 children with raised ICP at different time intervals the identified cut-off value was 4.3 mm for raised intracranial pressure with the sensitivity of 59.9% and specificity of 83.6%. By using Bayes nomogram, if the ONSD was more than the cut-off value (4.3 mm), the probability of high ICP was increased from 69 to 83%, and if the ONSD is less than 4.3 mm, the probability was decreased from 69 to 47%. Tayal et al. found that the possibility of raised ICP had dropped from 14 to 1% if ONSD was less than 5 mm and increased from 14 to 66% if ONSD was more than 5 mm [23]. Consequently, the ONSD is excellent method for detection of raised ICP.

The literature revealed varying cut-off values of ONSD in patients with raised ICP, each with a different sensitivity and specificity value. Rajajee et al. revealed a cut-off value of 4.8 mm for ICP > 20 cmHg [24]. Major et al. suggested 5 mm as a threshold limit for high ICP with 86% sensitivity and 100% specificity [25]. Teismann et al. identified a cut-off value of 4.6 mm which is consistent with a recent study by Yu ZY that found the ONSD cut-off value of 4.63 mm for elevated ICP [9, 26]. The interval between ocular sonography and the CT scan, which varied from 15 min to several hours or was not documented in numerous publications, may be a cause of this variability in a study. It is possible that this delay was the only methodological drawback that led to a significant amount of heterogeneity in the studies and caused the ICP to rise or fall between assessments [27]. In the current study CT was performed for all patients at a mean time of 1.35 h before admission and 2.21 h after admission, which was consistent with the findings of Goel et al. who reported the shortest time between CT and ONSD by ultrasonography [28]. Since treatment begins as soon as the patient is admitted to the PICU, a delay in the ONSD measurement from the CT scan may cause alterations in ICP. For example, early blood gas correction (PaCO2 and PaO2) may alter the ICP and, consequently, the ONSD evaluation, creating the false impression that the CT and ONSD results do not match. This is because blood gas correction will lower the ICP by at least 30% if there are no traumatic evidence for the elevated ICP [29].

In the current study, ONSD measurements were compared between children with opened and closed anterior fontanelles among the raised ICP group to study whether anatomical differences associated with fontanelle status might influence the transmission of intracranial pressure to the optic nerve sheath. We found that ONSD was significantly higher in the closed AF group on admission and one hour later. So, the patency of the anterior fontanelle may have an impact on ONSD. The open anterior fontanelle is believed to allow a degree of pressure compensation within the intracranial compartment, which could theoretically result in lower ONSD measurements despite elevated ICP. This hypothesis is supported by Padayachy et al. who stated that patency of the AF is a useful clinical marker for defining different ONSD cut-off values in children. [30]. Although we did not have direct ICP measurements for our patients, the comparison between open and closed AF groups was designed as a preliminary observational analysis to identify potential differences in ONSD values related to cranial anatomy. Further studies incorporating concurrent ICP monitoring are needed to validate the observed associations and determine the true influence of fontanelle status on ONSD.

The main objective of this study was the follow-up of ICP via ONSD among critically ill children. We studied changes in ONSD at four different time intervals for both raised and non-raised ICP groups, which showed significant decrease of ONSD in both groups. This may suggest that the absence of CT findings does not definitively rule out raised ICP. Also, the cutoff values of ONSD should be interpreted with caution in both raised and non-raised ICP groups categorized by CT imaging. The ONSD values dramatically dropped when the anti-ICP measures were administered. This coincided with the findings of Kerscher et al., in which 30 ICP-monitored patients showed significant decrease in ONSD following therapy for the elevated ICP [22]. In contrast, Cour-Andlauer et al. found that patients with increased ICP did not differ in their ONSD over the course of three days [31]. This might be due to including patients with serious brain injuries while in the current study we included only patients with non-traumatic causes of raised ICP.

## Limitations of the study

It is a single center study where only patients with non-traumatic causes of raised ICP were included. Despite prior sample size calculation, relatively small-sized population prohibited further stratification analysis, thus additional studies including non-traumatic causes of elevated ICP designed to categorize patients according to their medical conditions is recommended. Another limitation is the lack of gold standard invasive continuous methods for ICP monitoring as all patients underwent only non-invasive monitoring of ICP. Future studies involving invasive ICP monitoring to validate usage of ultrasound of ONSD in non-traumatic children is highly recommended.

## Conclusion

In critically ill children with non-traumatic causes of raised ICP on CT scan, point of care ultrasound of ONSD shows a good diagnostic test accuracy for evaluation of raised ICP as well as follow up tool of treatment response when used serially.

## Data Availability

The data used and/or analyzed during the current study are available from the corresponding author on reasonable request.
